# Visible and near-infrared spectroscopic method for correlative joint tissue analysis and visual and histologic grading

**DOI:** 10.1016/j.mex.2026.104085

**Published:** 2026-08-01

**Authors:** Amanda Spurri, Shu-Jin Kust, Alexandra Arnold, Petra Baylin, Nicholas Allen, Binyam Fentaw, Leslie Barnes, Daniela Proca, Nancy Pleshko

**Affiliations:** a Temple University, Department of Bioengineering, 1947 N. 12th Street, Philadelphia, PA, 19122, USA; b Lewis Katz School of Medicine Temple University, Department of Pathology and Laboratory Medicine, 3500 N. Broad Street, Philadelphia, PA, 19140, USA; c Lewis Katz School of Medicine, Temple University, Department of Orthopedic Surgery and Sports Medicine, 3500 N. Broad Street, Philadelphia, PA, 19140, USA

**Keywords:** Vibrational spectroscopy, Cartilage, Fiber optic probes, Musculoskeletal tissues, Histologic grading

## Abstract

Visible near-infrared (VNIR) spectroscopy is an analytical modality being investigated for non-destructive compositional assessment of musculoskeletal tissues, with recent work focused on development to enhance arthroscopic diagnostics. In this context, a primary objective is validation of the approach with histological grading which entails 1. Collection of hundreds of spectra from regions of joint cartilage in various degradation states followed by 2. Gold standard tissue histological grading, and 3. Correlative analysis of those data sets. A significant related challenge is spatial identification of regions of VNIR spectra collection from intact tissue segments and subsequent precision matching of those regions to guide locations for histological grading. Through establishing robust data sets, improved multivariate models can be developed for optical spectroscopic data-guided cartilage diagnostics.

The method described here proposes a strategy to support this essential aspect of data collection required to advance the VNIR modality for arthroscopic use and includes guidance for the following:

•Collection of VNIR spectra from human joint tissues after surgical tissue excision as part of arthroplasty surgery

•Association of collected spectra with tissue spatial location and identification of locations for histology grading

•Correlative multivariate analysis of VNIR spectra with corresponding histologic grade

## Graphical abstract

**Figure:**
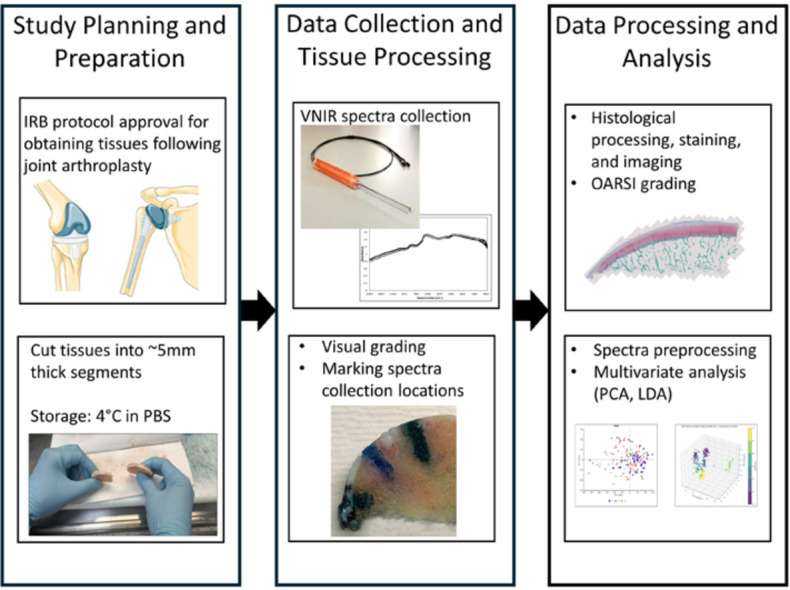

## Specifications table

**Table:** 

**Subject area**	Engineering
**More specific subject area**	Musculoskeletal diagnostics
**Name of your method**	VNIR Spectroscopic Data Acquisition Method for Joint Tissue Assessment and Comparisons to Visual and Histologic Grading
**Name and reference of original method**	Not applicable
**Resource availability**	VNIR Spectra Collection • Malvern Spectrometer: https://www.malvernpanalytical.com/en/products/product-range/asd-range/labspec-range • Fiber optic probe: https://shopstellarnet.com/reflectance-probe-vis-nir/ • Spectralon for background collection: https://www.edmundoptics.com/p/white-reflectance-standard-includes-99-standard/11120/?srsltid=AfmBOorEE2N-dR6xtyH3EScD5YyRJhUW1Y-G5tkipSpOMux0hQZ-PpQw Histology Reagents and Processing • Cal-Rite or Formical: https://www.mercedesscientific.com/epredia-richard-allan-scientific-cal-rite-decalcifying-fixation-solution • 10% Formalin: https://histologyconnections.com/10-formalin-fixative-neutral-phosphate-buffered/ • Sample inks for marking : https://www.statlab.com/sl662yl-2x.html • Safranin O stain: https://www.sigmaaldrich.com/US/en/substance/safraninosolution1234598765 • H&E stain: https://www.statlab.com/ds-h-e-stain-kitkthnept.html • Protease inhibitor: https://diagnocine.com/Product/Protease-Inhibitor-Cocktail-with-PBS-Tween-20-Buffer/95643?srsltid=AfmBOoqHNJf3WPtFrDG4TZ0K7NBQJuF4P1c2uh2MkDB9J3S0Q92b43oL • PBS: https://www.statlab.com/pbs-10x-concentrate-phosphate-buffered-saline-0–1m-pH-7-2bup0850.html Histology processing equipment: • Commercially available tissue processor: https://www.leicabiosystems.com/us/histology-equipment/tissue-processors/leica-tp1020/ • Commercially available embedding station system: https://www.leicabiosystems.com/us/histology-equipment/histology-embedding-centers-accessories/histocore-arcadia/ • Commercially available microtone: https://www.leicabiosystems.com/us/histology-equipment/microtomes/histocore-multicut/ • Microscope 10X and 20X objective / microscope slides • Software: • Unscrambler (Camo/AspenTech): https://www.aspentech.com/en/products/apm/aspen-unscrambler • Python: https://www.python.org/

## Background

Osteoarthritis (OA) is a musculoskeletal disorder where cartilage degradation and structural changes within joints can result in patient pain and reduced mobility. It is highly prevalent worldwide and with aging populations is expected to increase in the coming years [1]. Although arthroscopy is used as a surgical approach for both assessment and repair of joint injuries, these procedures do not consistently correlate with positive clinical outcomes [2,3]. Current arthroscopic diagnostics for cartilage evaluation include visual grading and tissue mechanical probing which have demonstrated limitations for reproducibility among surgeons [4,5]. These subjective techniques ultimately motivate the clinical need for more advanced arthroscopy diagnostic tools that can provide quantitative data.

**Fig. 1:**
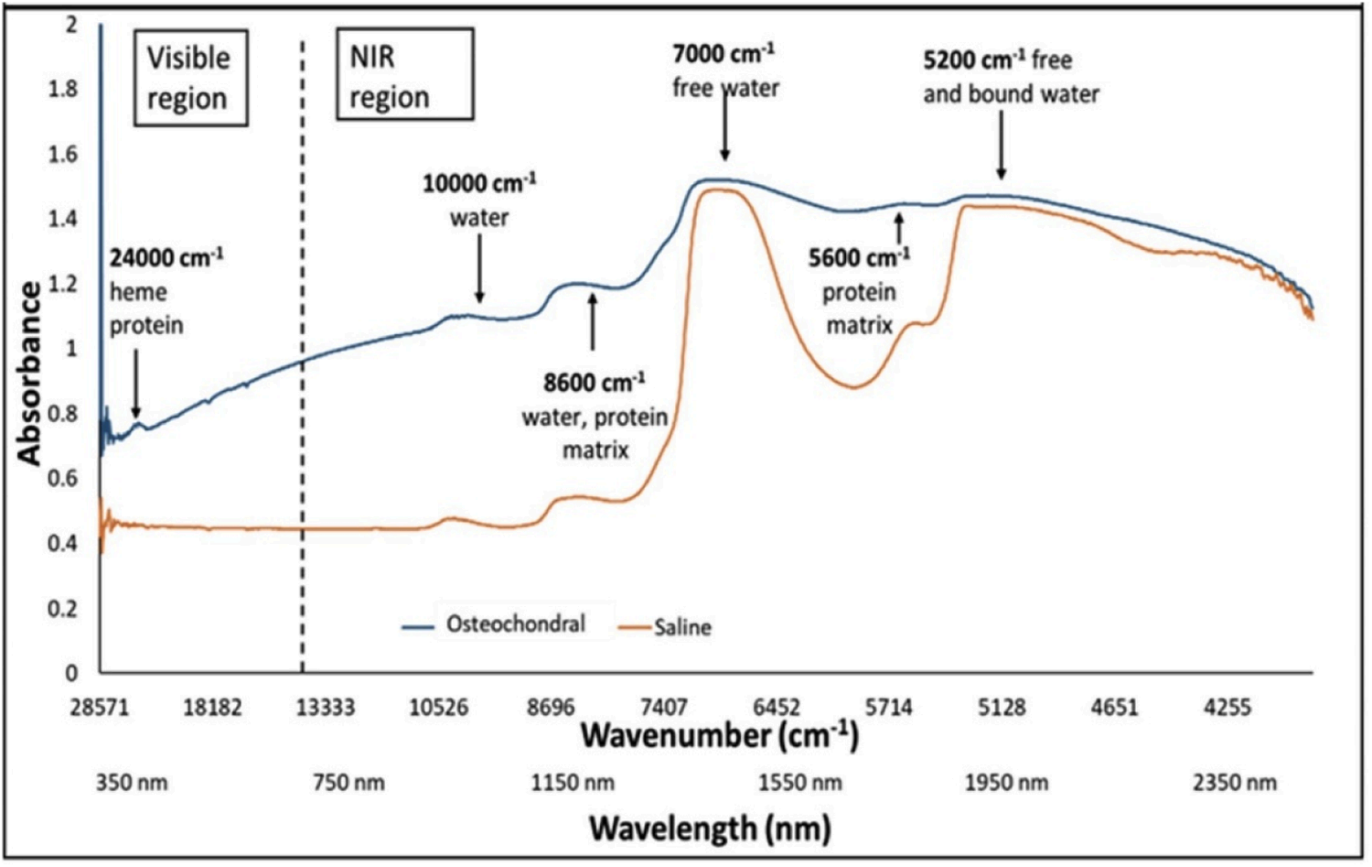
VNIR spectra of osteochondral tissue and saline showing absorbances related to water and protein in the NIR spectral region, and heme protein in the visible region. The X axis is shown in wavenumber and wavelength.

With the overarching goal of establishing methodology for arthroscopic assessment of musculoskeletal tissues, optical spectroscopic methods for joint tissue evaluation have been increasingly studied, including the application of Raman and Visible near-infrared (VNIR) fiber optic probes [[6], [7], [8]]. These fiber optic analytical methods are nondestructive and enable rapid acquisition of data, making them of high interest for evaluation of biological tissues. For VNIR spectra collection, molecular responses to light over the visible wavelengths (350 nm – 800 nm) and near-infrared (12500 cm^−1^ – 4000 cm^−1^) frequencies are utilized to assess compositional features of samples. Specifically for data collection from articular cartilage and joint tissues, spectral features correspond to the relative amounts of free and bound water [9], matrix proteins [10,11] and hemoglobin [12] (Fig. 1).

While optical analysis methods offer potential for compositional evaluation of musculoskeletal tissues, these techniques require validation with either biochemical analysis or gold standard histologic grading to progress development efforts. Validation with histology is advantageous as it is typically performed as a standard part of the post-surgical process. Histological grading of osteochondral joint tissues has been performed using several semiquantitative scoring systems, including the Osteoarthritis Research Society International (OARSI) [13], Mankin [14], and International Cartilage Repair Society II (ICRS-II) [15]. The Mankin score includes evaluation of several factors that change with joint degeneration, including cartilage structure, cellularity, matrix staining, and tidemark integrity, whereas the OARSI system primarily emphasizes the depth and extent of cartilage degeneration. Further, unlike the Mankin scoring system, the OARSI grading system accounts for reparative cartilage by incorporation of grading of advanced lesions containing fibrocartilaginous or regenerative repair tissue. This feature allows the OARSI system to distinguish reparative responses occurring in severe osteoarthritic lesions from complete cartilage loss. In contrast, the ICRS-II scoring system provides a comprehensive assessment of cartilage repair tissue, not degeneration, incorporating multiple features relevant to tissue-engineered or repaired cartilage. In this study, a modified OARSI grading system was used as it emphasizes the severity of structural cartilage degeneration and includes grading for reparative tissue response, and thus is well suited for evaluating progressive osteoarthritic change that occurs in end-stage OA.

While previous studies have demonstrated correlation of spectra to histologic grade in animal tissue samples [16] and human tissues [17,18], the methodology presented in this work stands to provide opportunities to utilize multiple tissue sites to expand capacity for spectral data acquisition. McGoverin et al. also collected NIR spectra from post-surgical osteochondral tissues, but locations of spectra collection were extracted with biopsy punches. In that study, spectra were collected from 35 total regions of the 22 tibial plateaus studied, less than two regions per tissue [17]. Alternatively, Tafintseva et al. collected spectra from extracted tissue plugs from 18 locations within cadaver knee joints, and samples were frozen and thawed prior to spectra collection [18]. While cadaver knee joints are effective for establishing the feasibility of spectral model development and the entire joint enables multiple locations for spectra collection, post-surgical tissues better reflect conditions encountered during arthroscopy. To expand the potential for model developments to support the high variability of human joint degradation, studies correlating spectra to histologic grade require large data sets collected from fresh tissues. There are several challenges associated with completing such studies, including gathering enough surgical samples and collecting data in a timely manner to avoid the need to freeze the tissue.

This method details instruction for collecting optical spectral data from multiple locations on individual fresh tissue segments so that the spatial locations can be associated with histologic grading and thickness measurements from deidentified tissues. Collecting spectra from multiple sites on one excised tissue section is beneficial for spectroscopic validation efforts because a range of degradation is typically present, enabling more complete utilization of tissue samples without additional cutting or freezing samples throughout the data collection process. As a result of the high variability and heterogeneity of the visual appearance of joint degradation in human tissue arthroscopy, robust models based on optical spectroscopic analysis techniques rely on establishment of comprehensive data sets. This method includes step by step details to ensure robust measurements that will ultimately be used as the basis for machine learning models to quantitatively and non-destructively assess cartilage tissue integrity.

## Method details

### Study information and preparation

The study was reviewed by the Temple University IRB (Protocol #31644) and determined not to be human subjects research. A survey template in Research Electronic Data Capture (REDCap), an application for storing and managing study data to maintain HIPAA protected data, was established for collecting surgical details of the tissues. The data captured as part of the survey included date of spectra collection, date of surgery, type of surgery, patient age, patient sex, number of locations for spectra collection, and visual grading score. Prior to study initiation, establishing a notification for surgery timeline and potential sample availability is critical to ensure that VNIR spectra are collected from samples within two days following the arthroplasty procedures.

### Sample collection and storage

Following routine surgical removal during joint arthroplasty for end-stage osteoarthritis from individuals age 41-80 years, including hip (n = 18), knee (n = 11) and shoulder (n = 5) replacements, samples were stored in phosphate-buffered saline (PBS) at 4 °C prior to VNIR spectra collection. Tissues utilized for this study did not include those with clinical suspicion of infection. Addition of protease inhibitor prevents protein matrix breakdown during sample storage. Prior to spectra collection, tissues were sliced into segments approximately 3 – 5 mm in thickness. For precision sectioning, a Marmed Band Bone Saw equipped with a water-cooled diamond blade was used. The water cooling minimized bone dust and aerosolization, while the 37-inch length diamond blade allowed for precise cuts of the specimen to the desired sizes. The optimal section thickness was approximately 3 mm, aligning with the diameter of the VNIR fiber optic probe tip.

### VNIR spectra collection

A custom design fiber optic probe was coupled with an ASD Labspec Four spectrometer (Malvern Panalytical). The fiber optic probe design included 6 illumination fibers and 1 collection fiber, all 300 µm in diameter. A commercially available VNIR probe can also be used. The fiber optic probe for this study incorporated a 90° bend at the tip containing a prism and glass spacer to direct the light around the bend, with a 3 mm tip diameter. Prior to spectra collection, the background was collected by placing the tip of the fiber optic probe in contact with a Spectralon standard (99% reflectance). The spectrometer software, Indico Pro (Malvern Panalytical) was configured so that 50 co-added scans were included per spectrum collected over the visible (350 – 800 nm (wavelength)) and near-infrared (12500 – 4000 cm^−1^(frequency)) regions with spectra obtained at 1 nm intervals. Any commercially available VNIR spectrometer and accompanying software can be used.

### Environmental and operational controls

For ex vivo tissue studies such as described in this Method, standard laboratory environments are appropriate for data collection, with temperature ranging from 20-25 °C and relative humidity 30-50%. The tissue should be removed from solution only immediately prior to spectral data collection to avoid dehydration, followed by gently dabbing with a Kimwipe to remove surface solution, and data collection completed within two to three minutes. The fiber optic probe tip should be cleaned between each tissue using an isopropyl alcohol pad (70 – 91%).

It is recommended to have the same user collect spectra throughout one experiment to standardize data collection, as variable contact with the tissue can impact spectra. A second option is to rely on the raw digital number (DN) signal output of the spectra at a specific frequency/wavelength that is not impacted by the absorbances of interest and is sensitive to tissue contact. This wavelength may be specifically chosen for the spectrometer/fiber optic. In the current experimental method setup, it was determined that the DN at 620 nm was sensitive to tissue contact. In this case, the concern is the potential to not be in contact with the tissue through air (as opposed to arthroscopy where the concern would be through fluid). In calibration experiments with intact osteochondral tissue, increasing the probe offset from direct contact to 0.5 mm resulted in an approximately 30% increase in the detected DN intensity at 620 nm. Increasing the offset to 1.0 mm produced an approximately 100% increase (∼twofold) in DN signal intensity relative to direct contact. This can be quantified for each individual probe and spectrometer and monitored prior to data collection. (As an aside, for arthroscopic environments with fluid, DN signal is decreased when the fiber optic is not in contact with the tissue compared to direct contact) [19].

### Sample and data nomenclature

Samples were stored in phosphate buffered saline (PBS) until just prior to spectra collection to maintain hydration. Once removed from PBS, spectra collection locations were identified on the tissue with the goal of finding a range of tissue degradation based on visual grade. Three spectra were collected per location to capture the maximum variability associated with data collection, as is common in spectroscopic studies. When saving a collected spectrum, a specific naming convention was used to ensure traceability to the associated location. An example of an appropriate naming convention is: 01-02-1, where the first label, 01, corresponds to the study patient number. The second label of the spectrum file name, 02, would correspond to the location number on the tissue, and the final label, 1, indicates the first out of three total spectra collected. The hospital patient identification was not involved in the spectra naming convention. For the purposes of the study, the samples were de-identified and numerical case numbers were substituted with the spectra naming convention (surgery numbers 1, 2, etc.). A research team member not involved with the spectral data collection and analysis kept the copy of the file with the patient case number and associated saved spectrum labels separate from the REDCap file.

### Visual grading at the time of VNIR spectra collection

At the time of spectra collection, locations were also assigned visual grades by two researchers based on the Collins scale [20], with a grade of 1 corresponding to visually intact cartilage and a grade of 4 corresponding to severely degraded cartilage. Photographs of the tissue segments were also taken so that they could be later referenced with histology sections. Visual grades were primarily used for identification of regions for data collection and not used for quantification. However, it is possible to use the visual data grading as part of a method where there is interest in understanding correlations between visual and histology grading.

### Tissue processing for histology

**Fig. 2:**
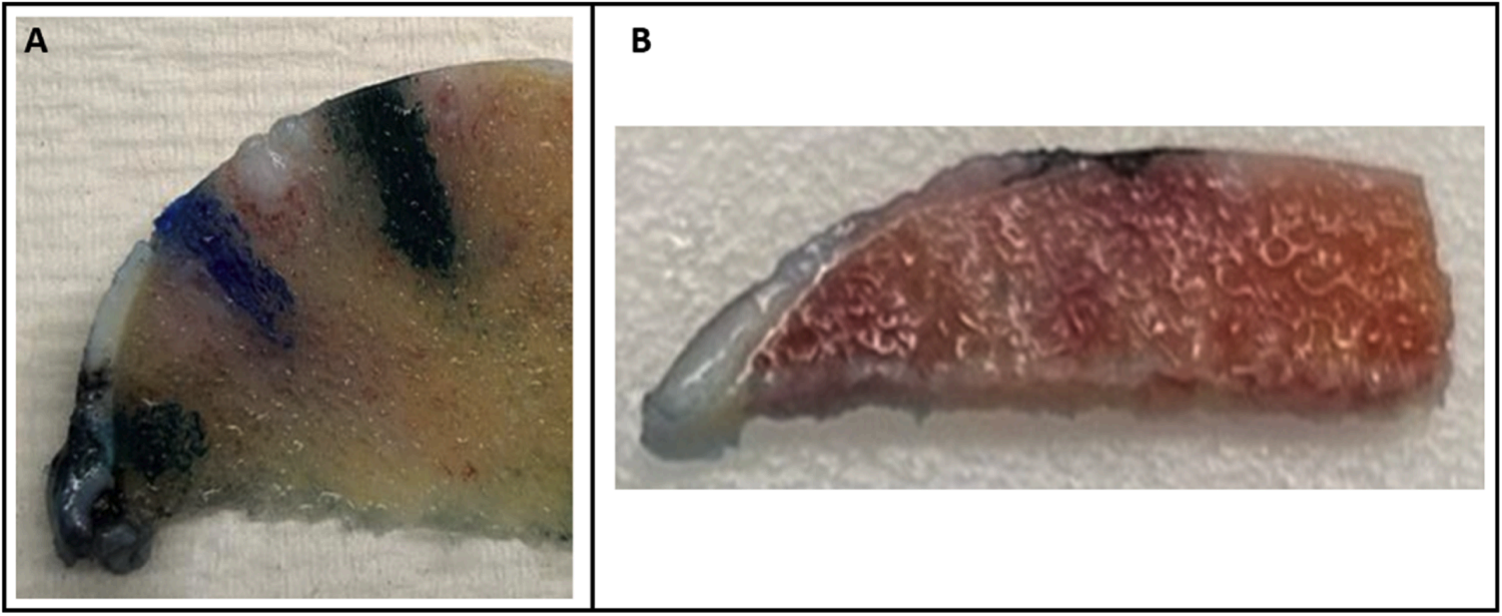
Photographs of tissue segments with histology markers indicating locations of VNIR spectra collection.

Immediately following spectra collection from each tissue segment region, locations were marked with a histology pen to identify the location of spectra collection. Multiple colors (black, blue, green) were utilized that would correspond to the location number on each tissue segment. For example, black ink would indicate the first location of spectra collection. The tissues were generally marked on the surface, but another option is to also mark into the bone for visualization (Fig. 2). Note: When using Safranin O/Fast Green staining for histological analysis, using colors other than green provides improved contrast from the histology stain.

The segments were fixed in 10% neutral buffered formalin and decalcified (Cal-Rite or Formical-2000). Following decalcification, the samples were embedded in paraffin (commercially available tissue processor and embedding center) and sectioned with a microtome at 5 µm thickness. Tissues sections were then stained separately with hematoxylin & eosin (H&E) and Safranin O/Fast Green (stains available from Sigma Aldrich). Stained slides were digitized for review using a microscope with 10X magnification.

### Histologic grading

Each location of spectra collection was graded according to the OARSI scale [13]. In this methodology, a grade of 0 corresponds to intact native cartilage and cartilage degradation increases with numerical score until grade 4.5. A grade of 5 indicates no remaining cartilage, and scores above 5 designate the presence of repair/regenerative tissue formation. Because the study objective was to categorize spectra based on ∼3 mm diameter spatial locations, a stage (horizontal extent of tissue impacted) was not included as part of the grading for this study. Histological sections were independently graded by three observers blinded to visual grades and spectral results using the modified OARSI scoring system. Prior to scoring the study tissues sections, the observers participated in a calibration session where they reviewed and discussed a representative set of sections spanning the full range of disease severity to establish consistent interpretation and application of the grading criteria. For the study samples, interobserver agreement was excellent, with an average pairwise quadratic weighted Cohen's kappa of 0.938 (range, 0.918–0.962).

### Thickness measurement

**Fig. 3:**
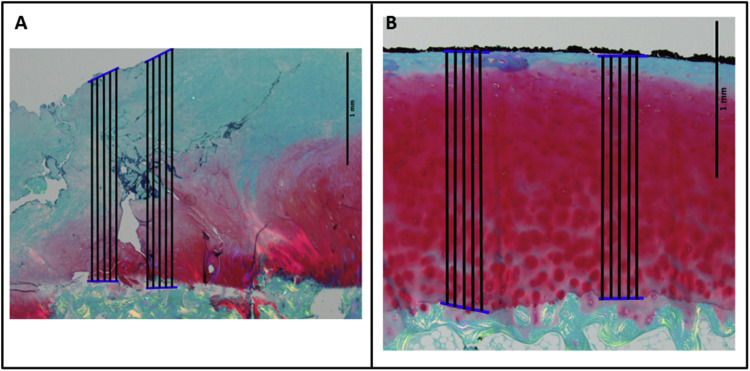
Microscopy images of Safranin O stained tissue sections with OARSI grades 2.5 (A) and 1.5 (B). Vertical black lines were used for thickness measurement.

Thickness measurements were taken to be used as a potential variable in future multivariate analysis, but were not incorporated into the results presented here. Thickness was measured on a subset of tissues from cartilage surface to the tidemark. As it was challenging to visualize the tidemark in some tissue sections, polarized light microscopy images were taken of the Safranin O stained samples on an Olympus BX53 polarized light microscope (Fig. 3). Based on the scale bars included in the images, ImageJ software was used to take measurements of selected tissues in the same regions where VNIR data were collected. Five equally spaced measurements were taken from two regions (10 measurements total per spectral collection region), and the ten measurements were averaged.

### Spectral processing and multivariate methods for correlative analysis

Spectra were initially pre-processed using the Unscrambler X software (Camo/AspenTech), and corresponding OARSI grades from a region of data collection were input as descriptive variables for categorizing or prediction. In general, there is no single “best approach” for pre-processing or for multivariate analysis, and for large data sets significant time is typically spent evaluating different analysis techniques. In the current study, spectral pre-processing techniques included extended multiplicative signal correction (EMSC) and derivative analysis [21]. Principal component analysis (PCA) and linear discriminant analysis (LDA) were evaluated as dimensionality reduction techniques to distinguish (separate) spectra based on the OARSI grade, not for independent prediction of grade. Wavelength/frequency ranges evaluated during PCA processing included 526 – 588 nm (19000 – 17000 cm^−1^) and 9200 – 4600 cm^−1^, investigating potential separation based on grade within the visible and NIR regions, respectively. Further, PCA was used as part of spectral outlier evaluation. PCA and Manhattan distance assessments were completed on the entire raw spectra set to indicate outliers for removal prior to additional spectral pre-processing.

LDA, a supervised dimensionality reduction and classification technique, seeks to maximize the separation between classes while minimizing within-class variance by projecting high-dimensional data onto a lower-dimensional space [22]. In spectroscopic applications, LDA is frequently implemented in combination with PCA (PCA-LDA) where PCA first reduces dimensionality to address collinearity among wavelengths (or frequencies) and potential singularity of the covariance matrix, followed by LDA for classification [23]. In the context of this study, LDA was used to explore whether spectra cluster according to histologic grade, rather than to develop a predictive classification model. For the broader spectral region (12,000 – 4000 cm^−1^), a standard linear discriminant analysis as implemented in scikit-learn (Python) was applied directly to the standardized spectral variables using the singular value decomposition solver. Although PCA-LDA, Partial Least Squares Discriminant Analysis (PLS-DA), and other variants of LDA are widely used in Vis-NIR and infrared spectroscopy, the optimal approach depends on sample size, degree of spectral collinearity, and whether the primary objective is classification, visualization, or interpretation. In this work, standard LDA was selected to generate low-dimensional discriminant scores that facilitate visualization of group separation and assessment of spectral clustering by OARSI grade.

The spectral processing techniques and multivariate analyses are common and further information can be found in reviews [[24], [25], [26]]. These analyses and outcomes will form the foundation for a larger scale machine learning model for prediction of histology grade from spectra.

### Method validation

**Fig. 4:**
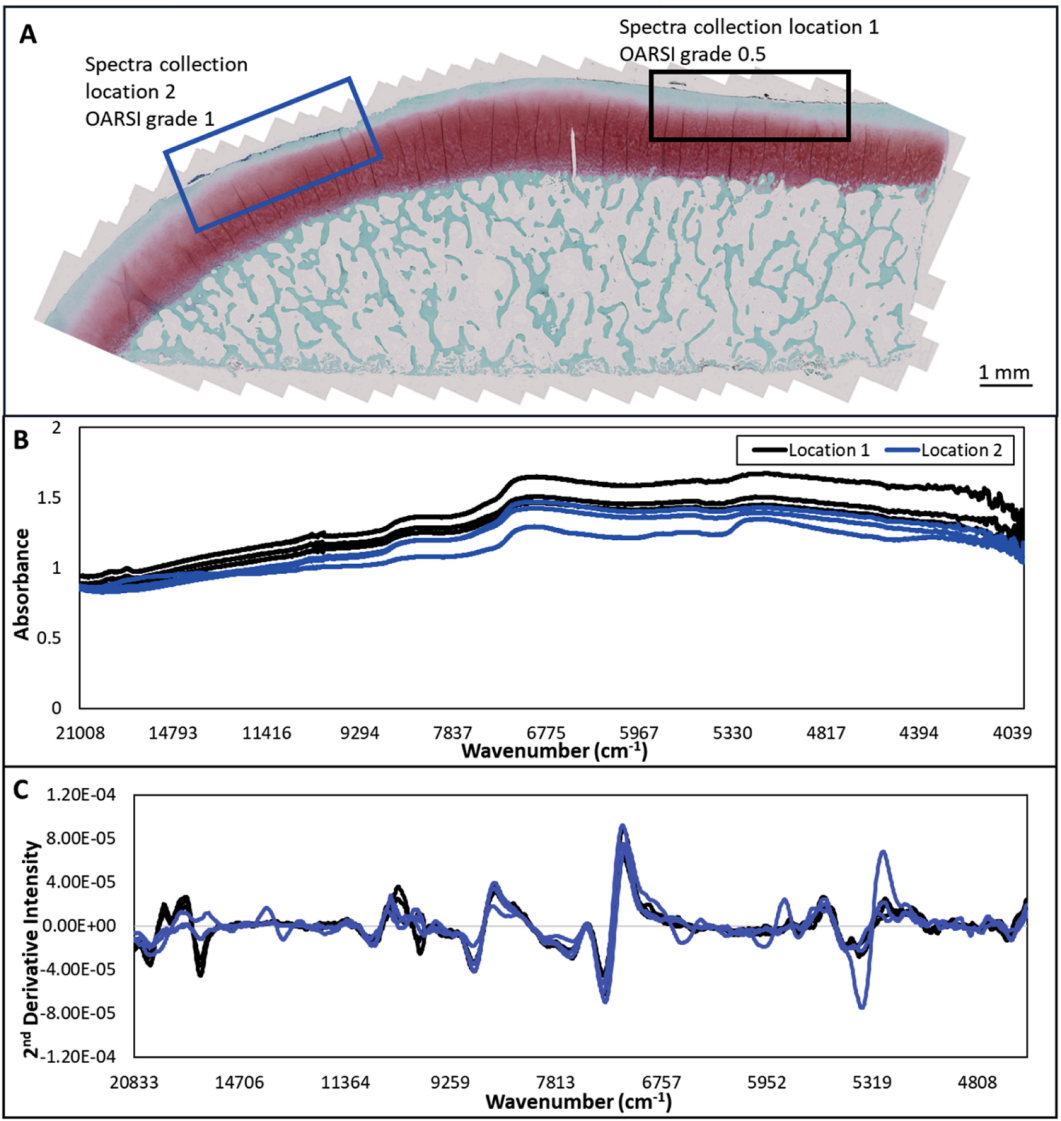
Safranin O stained histology image (A) with marked locations of spectra collection and corresponding raw spectra (B) and inverted second derivative spectra (Savitzky-Golay smoothing filter with 2nd order polynomial and 63 smoothing points) (C).

This study included spectra collection from 94 osteochondral tissue regions across 34 joint arthroplasty procedures from individuals that spanned the age range 41-80 years and included tissues from shoulder, hip, and knee joints. Using this method, experimental VNIR spectra were able to be matched to the associated collection location through identification of the histology ink on the sample surface (Fig. 4A). In this image, two individual locations on the sample tissue section were graded, enabling less obvious changes in the tissue to be assessed following VNIR spectra collection (Fig. 4B) and evaluation of the second derivative spectra for assessment of peak positions and relative intensities (Fig. 4C).

**Fig. 5:**
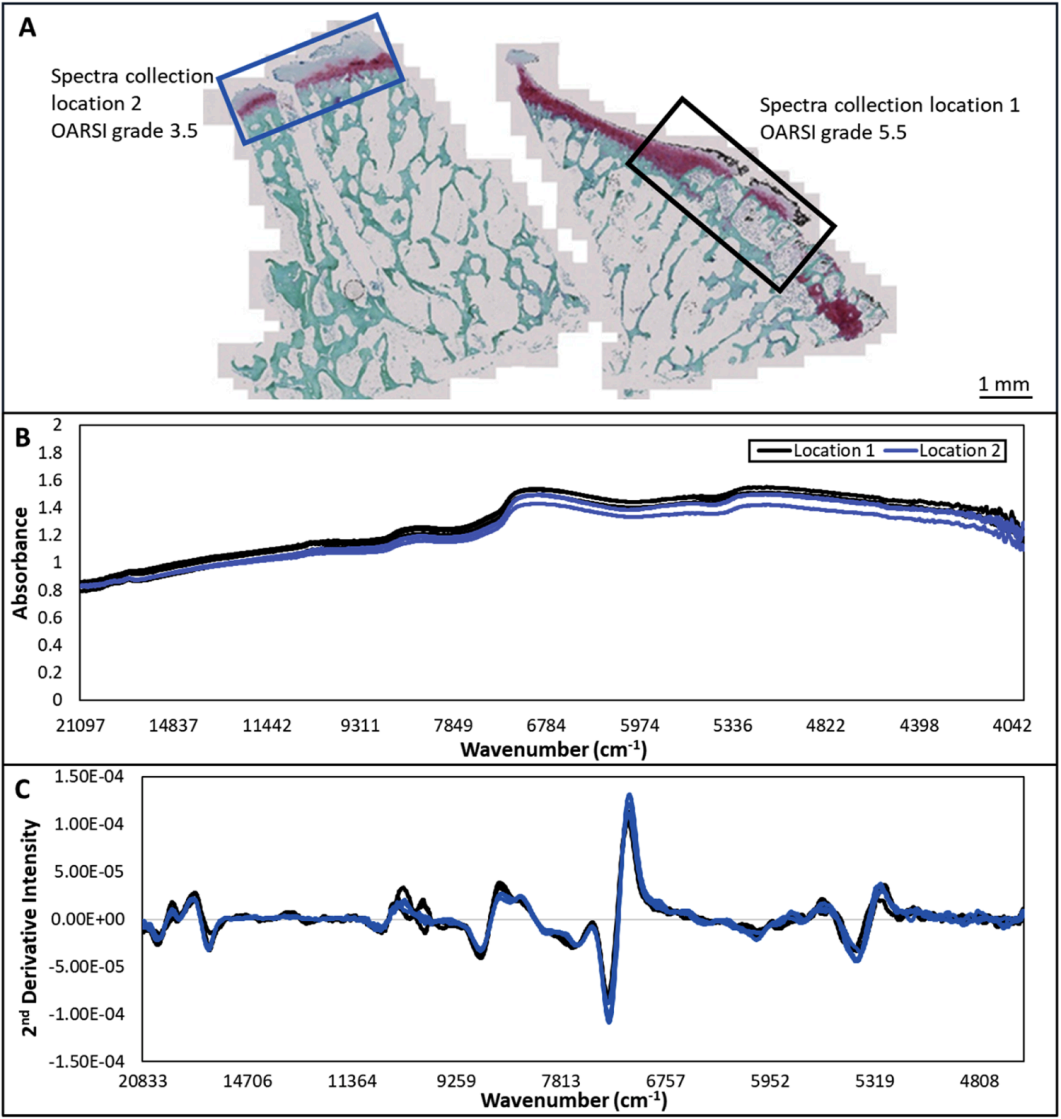
Representative image of (A) a tissue section stained with Safranin O with histology ink indicating spectra collection locations and corresponding raw spectra (B) and inverted second derivative spectra (Savitzky-Golay smoothing filter with *2*nd order polynomial and 63 smoothing points) (C).

While Fig. 4 shows a tissue sample with minimal cartilage degradation, spectra were collected from tissue with varying degrees of cartilage degradation and/or bone remodeling (Fig. 5A). Raw spectra (Fig. 5B) and inverted 2nd derivatives (Fig. 5C) demonstrate qualitative differences between the two locations of spectra collection.

**Fig. 6:**
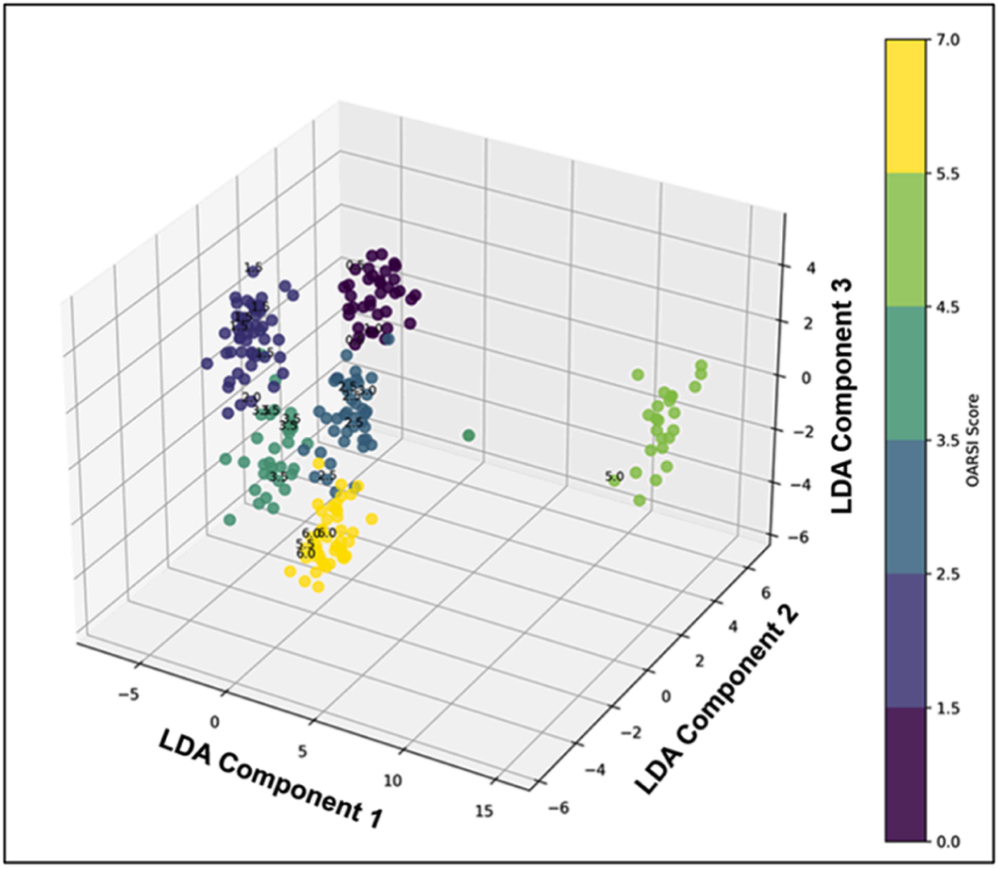
LDA projection of collected spectra as an exploratory visualization of spectral clustering by OARSI grade. Pre-processing spectral techniques included EMSC and second derivative using a Savitzky-Golay smoothing filter (2nd order polynomial and 33 smoothing points). It is important to note that LDA discriminant scores are dimensionless and thus there are no units on the axes.

The LDA analysis that used spectra pre-processed by EMSC and 2nd derivative Savitzky-Golay (2nd order polynomial with 33 smoothing points) in the region 12000 cm^−1^ – 4000 cm^−1^ provided results that demonstrated the ability to cluster spectra by histological OARSI grade, as shown in the LDA projection plot (Fig. 6). Even with the high variability of degradation, the OARSI grades are separated along component 3 from least tissue degradation to most tissue degradation with regenerative changes. Further the OARSI grade 5 indicates a tissue location containing bone tissue only, and this group is the most separated from the other groups on the LDA projection plot along component 1. With the addition of more spectra and grades from additional tissues, robust machine learning algorithms can be developed for independent prediction of histological grade. This will enable more precise surgical decisions regarding salvaging or removing tissue during arthroscopic procedures.

### Limitations of the method

Although this approach can be of widespread use in spectroscopic studies, there are several limitations to address. A primary limitation is the logistics involved in collecting spectra from tissues in a timely fashion, which can be challenging in a clinical hospital environment. Ideally a spectrometer would be available in the operating room such that spectra can be obtained immediately after tissue extraction. Further, for different optical spectroscopic techniques, it would be necessary to validate optimal positioning of the probe and tissue surface. Here, we have initiated studies to confirm contact with the tissue based on digital energy output changes at specific spectral frequencies, which is critical for the VNIR probe used in the current study [19].

Another limitation relates to the accuracy of marking tissues with histology pens. Three colors were used here, black, blue and green, to correspond to the three regions spectra were collected from on each tissue. However, the green was at times challenging to visualize given the tissue stain with fast green, and in approximately 20% of the tissue regions, the marker was not visible. Blue and black were always visible. However, at times the marker streaked across a larger region than anticipated, which made exact registration challenging in ∼ 10% of the samples. To minimize this happening, it is important to reduce excess fluid on the tissue surface.

We also note with respect to the histological grading, it is possible that a scoring system other than OARSI, such as Mankin, or perhaps a matrix grade only, may be optimal for correlations with VNIR spectra. Here, we are describing the methodological approach to ensure robust spectral data collection from a tissue site paired with a histology grade, without confirming which scoring system is best.

Despite these limitations, the proposed method has the potential to advance spectroscopic tissue compositional analysis by providing a standardized approach for acquiring multiple spatially distinct spectra from valuable surgical tissue specimens. The development of robust machine-learning models for spectroscopic tissue analysis is frequently constrained by the limited availability of well-characterized biological specimens, and many biomedical spectroscopy studies rely on relatively small cohorts, increasing the risk of overfitting and limiting model generalizability [27,28]. This challenge is particularly important to address for deep learning approaches, such as convolutional neural networks, which generally require substantially larger training datasets, often comprising hundreds of specimens, to achieve robust performance and avoid overfitting [29,30]. By enabling multiple independent spectral measurements to be obtained from a single tissue specimen while preserving tissue integrity for complementary analyses, the proposed method substantially increases the amount of spatially resolved spectral information that can be extracted from scarce surgical tissues. Consequently, this approach has the potential to facilitate the development of more robust machine-learning models and advance the state of the art in non-destructive spectroscopic tissue characterization.

## Ethics statements

This was not considered human subjects research (per Temple IRB committee).

## CRediT author statement

**Amanda Spurri:** Methodology, Investigation, Visualization, Writing – Original Draft, Reviewing and Editing

**Shu-Jin Kust:** Formal analysis, Investigation, Visualization, Writing – Reviewing and Editing

**Alexandra Arnold:** Formal analysis, Writing – Reviewing and Editing

**Petra Baylin:** Investigation, Visualization, Writing – Reviewing and Editing

**Nicholas Allen:** Methodology, Investigation, Writing – Reviewing and Editing

**Binyam Fentaw:** Methodology, Investigation, Writing-Reviewing and Editing

**Leslie Barnes:** Methodology, Supervision, Writing-Reviewing and Editing

**Daniela Proca:** Methodology, Supervision, Writing-Reviewing and Editing

**Nancy Pleshko:** Conceptualization, Supervision, Project administration, Writing- Reviewing and Editing

Supplementary material *and/or* additional information [OPTIONAL]

## Declaration of competing interest

The authors declare that they have no known competing financial interests or personal relationships that could have appeared to influence the work reported in this paper.

## Data Availability

Data will be made available on request.
